# Brief Communication: Maternal Plasma Autoantibodies Screening in Fetal Down Syndrome

**DOI:** 10.1155/2016/9362169

**Published:** 2016-03-06

**Authors:** Karol Charkiewicz, Monika Zbucka-Kretowska, Joanna Goscik, Slawomir Wolczynski, Adam Lemancewicz, Piotr Laudanski

**Affiliations:** ^1^Department of Perinatology and Obstetrics, Medical University of Bialystok, Marii Sklodowskiej-Curie 24a, 15-276 Bialystok, Poland; ^2^Department of Reproduction and Gynecological Endocrinology, Medical University of Bialystok, Marii Sklodowskiej-Curie 24a, 15-276 Bialystok, Poland; ^3^Faculty of Computer Science, Bialystok University of Technology, Wiejska 45A, 15-351 Bialystok, Poland

## Abstract

Imbalance in the metabolites levels which can potentially be related to certain fetal chromosomal abnormalities can stimulate mother's immune response to produce autoantibodies directed against proteins. The aim of the study was to determine the concentration of 9000 autoantibodies in maternal plasma to detect fetal Down syndrome.* Method.* We performed 190 amniocenteses and found 10 patients with confirmed fetal Down syndrome (15th–18th weeks of gestation). For the purpose of our control we chose 11 women without confirmed chromosomal aberration. To assess the expression of autoantibodies in the blood plasma, we used a protein microarray, which allows for simultaneous determination of 9000 proteins per sample.* Results.* We revealed 213 statistically significant autoantibodies, whose expression decreased or increased in the study group with fetal Down syndrome. The second step was to create a classifier of Down syndrome pregnancy, which includes 14 antibodies. The predictive value of the classifier (specificity and sensitivity) is 100%, classification errors, 0%, cross-validation errors, 0%.* Conclusion.* Our findings suggest that the autoantibodies may play a role in the pathophysiology of Down syndrome pregnancy. Defining their potential as biochemical markers of Down syndrome pregnancy requires further investigation on larger group of patients.

## 1. Introduction

The incidence of Down syndrome in the United States is estimated to be 1/732 live births [1]. This syndrome is a result of a chromosomal aberration characterized by extra chromosome 21 or a fragment thereof. In people with this aneuploidy, there is a high risk of congenital heart defects, gastroesophageal reflux syndrome, sleep apnoea, thyroid disease, and many other diseases [2].

Currently, the diagnosis of fetal Down syndrome is based on noninvasive (biochemical, genetic, and ultrasound) and invasive (amniocentesis and chorionic villous sampling) prenatal screening tests. Diagnostic efficacy of the invasive method in combination with genetic diagnostics is 99.8% and they rarely give false positive results. However, these methods carry a 1% risk of miscarriage or fetal damage [3]. A few years ago, scientists created a noninvasive prenatal test based on free fetal DNA (ffDNA) present in maternal blood. These tests have a low rate of false positives, which is only 0.5%, but they are still very expensive [4–7]. Therefore, there is a need for new potential biomarkers of Down syndrome pregnancy which will provide enough data for a small percentage of false positive results that will not have to be confirmed by any invasive method. Emerging evidence suggests that reproductive events and successful pregnancy outcome are under the regulatory control of cytokines and bioactive lipids, such as sphingolipids, but their role in human normal and abnormal pregnancies is still largely undefined [8–12]. The status of selected cytokines and sphingolipids in plasma and amniotic fluid of patients with chromosomally abnormal pregnancies has already been described [13, 14]. The current increased incidence of chromosomally abnormal pregnancy loss could depend on the aneuploidy that correlates with a disturbance of the release of some cytokines of placental perfusion and uterine contraction. The imbalanced levels of inflammatory cytokines in the case of abortion, preterm labour, premature rupture of the membranes, and fetal inflammatory response syndrome, where infection is absent, could be interpreted as a consequence of a genetic feature that results in fetus participating in the mechanism of its own distress, death, and expulsion [8]. Moreover, one of the more recent publications revealed that most of the deregulated genes (in Down syndrome) were involved in “angiogenesis,” “inflammation mediated by cytokines and chemokines,” “integrins,” and “interleukins” signaling pathways, all of which can potentially lead to abnormal secretion of different molecules into mothers circulation [9]. It can be suggested that significant imbalance in the levels of different circulating metabolites in maternal blood can stimulate mother's immune response to produce autoantibodies directed against the abovementioned proteins. Therefore, measuring the expression of autoantibodies in pregnancies with fetal chromosomal abnormalities could lead to better understanding of the influence of Down syndrome on such pregnancy and possibly provide new biomarker(s) for noninvasive genetic testing.

## 2. Material and Methods

The study and control groups consisted of women who underwent routine amniocentesis between 15th and 18th week of gestation at the Department of Reproduction and Gynecological Endocrinology of the Medical University of Bialystok, Poland (recruitment between September 2012 and October 2013). We performed 190 amniocenteses throughout the recruitment period. We included only nonfebrile women without any chronic or acute diseases and excluded women taking any type of hormonal or anti-inflammatory treatment as well as those with vaginal and urinary tract symptoms that would suggest infection. We also excluded all pregnant women with previously diagnosed autoimmune diseases or with these diseases in their family history.

The study protocol was approved by the Local Ethics Committee of Medical University of Bialystok (Poland) (Approval number: R-I-002/36/2014). Signed informed consent was obtained from all participants involved in the study.

We collected 10 mL of peripheral blood into EDTA tubes from each patient after successfully performed amniocentesis. The blood was then centrifuged, plasma subsequently separated, and frozen at −80°C temperature. After analyzing karyotype testing results, we chose 10 women with trisomy 21 fetuses into the study group and selected 11 healthy patients with uncomplicated pregnancies, who delivered healthy newborns at term for the control group.

To assess the expression of autoantibodies in the blood plasma we used the ProtoArray® Human Protein Microarray 5.1 (Invitrogen, USA), which allows for simultaneous determination of 9000 proteins per sample. This microarray was the first high-density microarray and it contains thousands of unique, full-length human proteins including kinases, phosphatases, GPCRs, nuclear receptors, and proteases, spotted in duplicate on a thin nitrocellulose coated glass slide with thickness 1 inch × 3 inches. ProtoArray Human Protein Microarray version 5.1 contains over 9000 unique human proteins individually purified and arrayed under native conditions to maximize functionality.

A capture protein was first bound to a glass surface. After incubation with the sample, the target antibody was trapped on a solid surface. A second biotin-labeled detection antibody was then added, which can recognize a different isotope of the target autoantibody. The protein-autoantibody-antibody-biotin complex was then visualized through adding Streptavidin-Alexa Fluor® 647 Conjugate and viewing with a laser scanner (GenePix 4100A). We also evaluated plasma C-reactive protein (CRP) levels using immunoturbidimetric method with the Multigent CRP Vario assay (detectable range was 0.2–480 mg/L) detected on the ARCHITECT ci4100.

Computer analysis aiming at discovering proteins whose expression significantly differs in defined groups was performed using the Bioconductor limma package [15]. Preprocessing data with background correction and between-array normalization was the first step of the analysis. The purpose of this step was to transform the original data to enable comparing the results of multiple experiments (21 microarrays), obtaining approximate protein expression distribution across all of the arrays. We performed background correction using the normexp method [16], whereas for between-array normalization we applied the quantile method [17]. We determined the proteins undergoing statistically significant differential expression in the compared groups by fitting multiple linear models with the generalized least squares fitting method. Subsequently, we used the empirical Bayes method to rank the proteins in order of evidence for differential expression [18]. Significance level (alpha) equal to 0.05 and minimal absolute value of logged fold change (logarithm base 2) equal to 0.5 were fixed for all calculations. As the next step of the analysis, we validated the classification capability of the previously chosen proteins, showing differential expression and treated as features. Considering high probability of occurrence of similar expression profiles between the selected proteins, we used a feature selection procedure with the tools provided by the caret package [19]. Pearson correlation coefficient equal to at least 0.5 (in its absolute value) was taken as a threshold for considering features to be significantly correlated. After eliminating redundant features, we checked the classification accuracy of the remaining features using the Support Vector Machines classifier with the radial basis (Gaussian) kernel function and leave-one-out cross-validation procedure. The threshold value of the correlation coefficient was chosen to obtain the best classification accuracy with the smallest possible number of features. Features were standardized to zero mean and unit variance. Kernlab package [20] was employed for classification and validation. All of the computer analyses were conducted using the R software environment [21].

## 3. Results

Clinical characteristics of the patients are presented in Table 1. Statistical analysis of the expression of 9000 autoantibodies revealed that the expression of 213 autoantibodies (Table 2) is statistically significantly different (decreased or increased) when comparing the group with fetal Down syndrome and the control group. The next step of the analysis was to create a classifier providing the best possible discrimination between the studied groups. After eliminating redundant variables, as described in the previous section, 14 autoantibodies (Table 3) were chosen for further investigation. To test their predictive capability we built the Support Vector Machines classifier using the selected autoantibodies as features. The classification accuracy equal to 100% (i.e., cross-validation error equal to 0%) was obtained using the leave-one-out cross-validation technique and treating the selected autoantibodies as features.

The classifier is a set of autoantibodies whose concentrations do not correlate with each other, since each protein is independent of the other. These proteins together have greater sensitivity and specificity than each of them separately. Based on this set, it could be possible to create, in the future, a special software to estimate the risk of fetal Down syndrome by analyzing the concentrations of these autoantibodies in the mother's blood.

We did not find any statistically significant differences when we compared the plasma CRP concentrations between the study and control groups using Wilcoxon rank-sum test.

## 4. Comment

It is difficult to compare the results of our investigation to any other research, because of the lack of any articles about autoantibodies' profiling in maternal blood plasma of patients with fetal chromosomal abnormalities. Nevertheless, it is possible to associate some information available in the literature with our study results. There are potential explanations for the role of differentially expressed antibodies in the pathophysiology of Down syndrome pregnancy.

It is becoming more and more commonly acknowledged that fetal chromosomal aberration can cause imbalance in the metabolites levels in maternal blood. A number of studies describe inflammatory factors, hormones, and lipids potentially related with trisomy 21 [8, 9, 13, 14]. Hence, our hypothesis is that significant changes in the blood metabolites profile of pregnant women diagnosed with fetal Down syndrome can stimulate mother's immune system and consequently lead to abnormal production of autoantibodies to maternal blood. The results of our investigation seem to confirm this hypothesis.

Initially, we compared the expression of all autoantibodies between the study and the control group. We revealed 213 statistically significant autoantibodies, whose expression decreased or increased in the group with fetal Down syndrome in comparison to the control group. Among these 213 proteins there were autoantibodies directed against well-known and described proteins in Down syndrome, for example, lamin-A/C [22], interleukin-1 receptor-associated kinase-like 2 [23], interleukin 17C [24], aminoadipate aminotransferase [25], calcium/calmodulin-dependent protein kinase kinase 1 [26], septin 4 (transcript variant 1) [27], serine/threonine kinase [28], albumin [29], elastase 2B [30], glycine N-methyltransferase [31], N-ethylmaleimide-sensitive factor attachment protein, gamma [32], dynamin 2 [33], tropomodulin-2 [34], interleukin-1 alpha [35], and selectin P ligand [36]. This finding may indirectly confirm the accuracy of our research. However, we believe that the classifier described in the present study is more interesting than just comparing individual autoantibodies. The classifier is of high diagnostic value and it indicates a potential new way of diagnosing fetal Down syndrome. The limitation of the study is a relatively small study group, but this is only a preliminary experiment and the results should be confirmed in a larger study population. In our next experiment, we expect to obtain enough high specificity and sensitivity of our classifier to eliminate the necessity of confirming the results by invasive methods.

From our study we excluded patients with symptoms of inflammation (only nonfebrile patients with negative CRP plasma levels were included in the study), which allows us to suspect that fluctuations of the autoantibodies' expression may be the result of fetal chromosomal aberration. Another limitation of the study is the lack of white blood count results; however, they are not routinely performed before each amniocentesis.

In the present study, we showed that selected autoantibodies could be potential biomarkers of Down syndrome pregnancies and could play a role in the pathology of trisomy 21. In the available literature there is still no relevant research focused on the role of autoantibodies in the pathogenesis of Down syndrome pregnancies. Therefore, it is difficult to definitely conclude on the variations in the levels of autoantibodies. However, due to the complexity of the pathomechanism responsible for fetal Down syndrome, further functional experiments should be performed.

## Figures and Tables

**Table 1:** Clinical characteristic of the patients.

	Group I, Down syndrome pregnancies (*n* = 10)	Group II, pregnancies without Down syndrome (*n* = 11)
Maternal age (median ± SD)	39.5 ± 8.193	38 ± 8.799
Number of pregnancies (median ± SD)	1.5 ± 0.9189	1 ± 1.168
Gestational age at collecting of samples in weeks (median ± SD)	15.85 ± 0.7633	16.8 ± 1.048

SD: standard deviation.

**Table 2:** The 213 statistically significant autoantibodies, whose expression decreased or increased in the group with fetal Down syndrome in comparison to the control group.

	Name of autoantibody: antibody directed against the following proteins	Log FC (if there is negative value, it is decreased autoantibody expression in Down syndrome group versus control group; if there is positive value, it is increased autoantibody expression in Down syndrome group versus control group)	*P* value
1	Recombining binding protein suppressor of hairless (*Drosophila*) (RBPSUH), transcript variant 3, mRNA	1,60	0,00
2	Hematological and neurological expressed 1 (HN1), transcript variant 3	1,55	0,01
3	Hepatitis B virus x interacting protein (HBXIP)	1,54	0,02
4	Recombination signal binding protein for immunoglobulin kappa J region (RBPJ), transcript variant 4	1,45	0,00
5	Alcohol dehydrogenase, iron containing 1 (ADHFE1)	1,41	0,01
6	Transcription factor CP2-like 1 (TFCP2L1)	1,39	0,01
7	WW domain containing oxidoreductase (WWOX), transcript variant 3	1,33	0,03
8	Angiogenin, ribonuclease, RNase A family, 5, mRNA (cDNA clone MGC:61969 IMAGE:6453640), complete cds	1,28	0,01
9	Ephrin receptor B1 (EPHB1)	1,26	0,01
10	Spi-C transcription factor (Spi-1/PU.1 related) (SPIC)	1,22	0,01
11	SUMO1 activating enzyme subunit 2 (SAE2)	1,15	0,02
12	Family with sequence similarity 108, member B1 (FAM108B1)	1,11	0,00
13	SFRS protein kinase 1 (SRPK1)	1,04	0,02
14	FGF6 recombinant human protein	1,03	0,04
15	BTB/POZ domain containing protein KCTD18	1,01	0,03
16	Zinc finger CCHC domain containing protein 8	1,00	0,04
17	Mediator of RNA polymerase II transcription subunit 22	0,99	0,01
18	Minichromosome maintenance complex component 2 (MCM2)	0,99	0,03
19	ANKRD26-like family B member 1	0,98	0,00
20	Casein kinase 2, alpha prime polypeptide (CSNK2A2)	0,97	0,01
21	Lectin, Galactoside-Binding, Soluble, 14 (LGALS14), transcript variant 2	0,95	0,04
22	Stress 70 protein chaperone, microsome-associated, 60 kDa (STCH)	0,94	0,00
23	Suppressor of Ty 4 homolog 1 (*S. cerevisiae*) (SUPT4H1)	0,94	0,00
24	Ephrin type-B receptor 2	0,93	0,01
25	WD repeat domain 69 (WDR69)	0,92	0,02
26	Chromosome 6 open reading frame 206 (C6orf206)	0,92	0,02
27	v-akt murine thymoma viral oncogene homolog 1 (AKT1), transcript variant 3	0,91	0,04
28	Surfeit 5 (SURF5), transcript variant a	0,90	0,01
29	Calcium/calmodulin-dependent protein kinase (CaM kinase) II alpha (CAMK2A), transcript variant 1	0,90	0,01
30	P antigen family, member 2 (prostate associated) (PAGE2)	0,88	0,03
31	Acyl-coenzyme A binding domain containing 7 (ACBD7)	0,88	0,03
32	Chromosome 18 open reading frame 32 (C18orf32)	0,87	0,04
33	mRNA similar to oocyte-specific histone H1 (cDNA clone MGC:50807 IMAGE:5742122), complete cds	0,86	0,04
34	Zinc finger protein SBZF3, mRNA (cDNA clone MGC:14334 IMAGE:4298348), complete cds	0,84	0,01
35	Protein DDI1 homolog 1	0,84	0,00
36	Proline-rich transmembrane protein 2 (PRRT2)	0,83	0,05
37	Mitogen-activated protein kinase kinase kinase 7 (MAP3K7), transcript variant B	0,82	0,04
38	Kv channel interacting protein 4 (KCNIP4), transcript variant 1	0,81	0,04
39	Nucleoredoxin	0,79	0,01
40	Hypothetical protein MGC40069 (MGC40069)	0,78	0,02
41	Chemokine (C-X-C motif) ligand 10, mRNA (cDNA clone MGC:13622 IMAGE:4274617), complete cds	0,77	0,04
42	Zinc finger, matrin type 5 (ZMAT5), transcript variant 1	0,77	0,00
43	Parvin, alpha (PARVA)	0,75	0,05
44	Interleukin-6	0,75	0,05
45	Eukaryotic elongation factor-2 kinase	0,75	0,04
46	Prefoldin subunit 4 (PFDN4)	0,74	0,02
47	Hypothetical protein FLJ10986 (FLJ10986)	0,74	0,03
48	Hypothetical protein MGC3020 (MGC3020)	0,73	0,03
49	Heat shock factor binding protein 1 (HSBP1)	0,73	0,02
50	Mitogen-activated protein kinase kinase kinase kinase 2 (MAP4K2)	0,72	0,03
51	Hypothetical protein MGC24103 (MGC24103)	0,72	0,02
52	Chromosome 7 open reading frame 36 (C7orf36)	0,72	0,05
53	Forkhead box P3 (FOXP3)	0,72	0,04
54	Ephrin receptor A1 (EPHA1)	0,71	0,02
55	ELL associated factor 1 (EAF1)	0,71	0,02
56	Exosome component 8 (EXOSC8)	0,70	0,02
57	Sialidase 4 (NEU4)	0,70	0,02
58	Activating signal cointegrator 1 complex subunit 2 (ASCC2)	0,70	0,03
59	Chemokine (C-C motif) ligand 13 (CCL13)	0,69	0,00
60	DNA-directed RNA polymerases I and III subunit RPAC1	0,69	0,01
61	Septin 4 (SEPT4), transcript variant 1	0,69	0,03
62	*α* serine/threonine kinase	0,69	0,02
63	Protein tyrosine phosphatase, receptor type, O (PTPRO), transcript variant 3	0,68	0,01
64	Nudix (nucleoside diphosphate linked moiety X) type motif 2 (NUDT2), transcript variant 1	0,68	0,03
65	Protein phosphatase 1, regulatory (inhibitor) subunit 2 pseudogene 9 (PPP1R2P9)	0,68	0,03
66	Septin 4 (SEPT4), transcript variant 3	0,68	0,04
67	Nuclear receptor coactivator 5	0,68	0,03
68	WD repeat domain 53 (WDR53)	0,67	0,02
69	RAR-related orphan receptor B (RORB)	0,67	0,00
70	Chromosome 8 open reading frame 22 (C8orf22)	0,66	0,02
71	Chromosome 21 open reading frame 25 (C21orf25)	0,64	0,02
72	Albumin (ALB)	0,64	0,03
73	Chromosome 10 open reading frame 83 (C10orf83)	0,63	0,01
74	StAR-related lipid transfer (START) domain containing 10 (STARD10)	0,63	0,04
75	Minichromosome maintenance complex component 7 (MCM7)	0,62	0,04
76	Elastase 2B (ELA2B)	0,62	0,04
77	WD repeat domain 5B (WDR5B)	0,61	0,02
78	Exosome component 5 (EXOSC5)	0,61	0,04
79	Spleen focus forming virus (SFFV) proviral integration oncogene spi1 (SPI1), mRNA	0,59	0,04
80	fms-related tyrosine kinase 3 ligand (FLT3LG)	0,59	0,03
81	Hemoglobin, gamma A (HBG1)	0,59	0,03
82	Leukocyte-associated immunoglobulin-like receptor 2 (LAIR2), transcript variant 1	0,59	0,05
83	Forkhead box P1 (FOXP1)	0,58	0,03
84	Polymerase (DNA-directed), delta 4 (POLD4)	0,58	0,04
85	Hypothetical protein AL133206 (LOC64744), mRNA	0,58	0,02
86	Ubiquitin-conjugating enzyme E2L 6 (UBE2L6), transcript variant 1	0,57	0,03
87	Protein kinase C, beta 1 (PRKCB1), transcript variant 2	0,57	0,04
88	M-phase phosphoprotein 6 (MPHOSPH6)	0,57	0,01
89	Zinc finger protein 765 (ZNF765)	0,56	0,01
90	FtsJ homolog 1 (*E. coli*) (FTSJ1), transcript variant 1	0,56	0,04
91	Ring finger protein 128 (RNF128), transcript variant 1	0,56	0,02
92	TNFRSF1A/TNFRI/CD120a protein (His Tag)	0,55	0,05
93	Acid phosphatase 6, lysophosphatidic (ACP6)	0,55	0,02
94	Nucleophosmin (nucleolar phosphoprotein B23, numatrin) (NPM1)	0,55	0,02
95	Kelch domain containing 3 (KLHDC3), mRNA	0,55	0,03
96	N(6)-Adenine-specific DNA methyltransferase 1	0,55	0,05
97	RAB4A, member RAS oncogene family (RAB4A)	0,54	0,03
98	Zinc finger protein 396 (ZNF396), mRNA	0,54	0,02
99	kinesin family member 3A (KIF3A)	0,53	0,04
100	Poly(rC)-binding protein 2	0,53	0,05
101	WD repeat and FYVE domain containing 3 (WDFY3), transcript variant 3	0,53	0,05
102	Glycine N-methyltransferase (GNMT)	0,53	0,01
103	Histone H2B type 1-H	0,53	0,04
104	Tumor necrosis factor, alpha-induced protein 8-like 1 (TNFAIP8L1)	0,52	0,02
105	BRCA2 and CDKN1A interacting protein (BCCIP)	0,52	0,02
106	DnaJ (Hsp40) homolog, subfamily B, member 11 (DNAJB11)	0,52	0,05
107	Lamin-A/C	0,51	0,04
108	Seven in absentia homolog 1 (*Drosophila*) (SIAH1), transcript variant 2, mRNA	0,50	0,05
109	Ninjurin 2 (NINJ2)	−0,50	0,04
110	Trypsin-2	−0,50	0,02
111	PREDICTED (uORF:IOH62458~RFU:1604.5)	−0,51	0,04
112	Chromosome 20 open reading frame 39 (C20orf39)	−0,52	0,03
113	Dual specificity mitogen-activated protein kinase kinase 3	−0,52	0,04
114	Polymerase (RNA) III (DNA directed) polypeptide C (62 kDa) (POLR3C)	−0,52	0,03
115	Interleukin-1 receptor-associated kinase-like 2	−0,52	0,02
116	Adenylate kinase 2 (AK2), transcript variant AK2A	−0,53	0,05
117	pim-3 oncogene (PIM3)	−0,53	0,04
118	Chromosome 20 open reading frame 71 (C20orf71)	−0,53	0,04
119	LSM12 homolog (*S. cerevisiae*) (LSM12)	−0,54	0,03
120	Ring finger and CHY zinc finger domain containing 1 (RCHY1)	−0,54	0,02
121	Carbonic anhydrase X (CA10)	−0,55	0,02
122	Phosphoglucomutase 2-like 1 (PGM2L1)	−0,55	0,02
123	Membrane-associated ring finger (C3HC4) 10 (RNF190)	−0,56	0,05
124	Fructose-1,6-bisphosphatase 1 (FBP1)	−0,56	0,05
125	Myotubularin related protein 8 (MTMR8)	−0,57	0,02
126	Transient receptor potential cation channel subfamily M member 3	−0,57	0,04
127	ATP citrate lyase (ACLY)	−0,57	0,03
128	TNFSF10/APO2L/TRAIL/CD253 protein (native)	−0,58	0,04
129	Hypothetical protein FLJ33008 (FLJ33008), mRNA	−0,58	0,04
130	Proline rich 14 (PRR14)	−0,58	0,02
131	Interleukin 17C (IL17C), mRNA	−0,58	0,01
132	Upstream stimulatory factor 2	−0,58	0,02
133	Procollagen C-endopeptidase enhancer 1	−0,59	0,02
134	Thymine-DNA glycosylase (TDG)	−0,59	0,05
135	Matrix metallopeptidase 7 (matrilysin, uterine) (MMP7), mRNA	−0,59	0,04
136	DSN1, MIND kinetochore complex component, homolog (*S. cerevisiae*) (DSN1)	−0,60	0,02
137	PTK6 protein tyrosine kinase 6 (PTK6)	−0,60	0,05
138	Tubulin tyrosine ligase-like family, member 6 (TTLL6)	−0,60	0,01
139	Spastic paraplegia 21 (autosomal recessive, mast syndrome) (SPG21)	−0,61	0,04
140	Forkhead box M1, clone MGC:10704 IMAGE:3833837, mRNA, complete cds	−0,61	0,04
141	Embigin homolog (mouse) (EMB)	−0,62	0,04
142	Dynamin-2	−0,63	0,01
143	Mitogen-activated protein kinase-activated protein kinase 3 (MAPKAPK3)	−0,64	0,03
144	Runt-related transcription factor 1, translocated to 1 (cyclin D-related) (RUNX1T1), transcript variant 1	−0,64	0,04
145	Carnitine O-acetyltransferase	−0,64	0,01
146	Cell division cycle 25 homolog C (*S. pombe*) (CDC25C), transcript variant 1	−0,65	0,02
147	Menage a trois homolog 1, cyclin H assembly factor (*Xenopus laevis*) (MNAT1)	−0,65	0,01
148	Obg-like ATPase 1 (GTPBP9)	−0,65	0,03
149	Rho GTPase activating protein 24 (ARHGAP24), transcript variant 2	−0,65	0,01
150	abl-interactor 1 (ABI1)	−0,66	0,05
151	Uncharacterized protein C6orf81	−0,66	0,05
152	1-Aminocyclopropane-1-carboxylate synthase-like protein 1	−0,67	0,02
153	Rho GTPase activating protein 12	−0,68	0,04
154	Mitogen-activated protein kinase kinase 3 (MAP2K3), transcript variant B	−0,68	0,00
155	Aminoadipate aminotransferase (AADAT)	−0,69	0,02
156	DCP1 decapping enzyme homolog B (*S. cerevisiae*), mRNA (cDNA clone MGC:44405 IMAGE:5296928), complete cds	−0,69	0,02
157	Calcium/calmodulin-dependent protein kinase kinase 1	−0,69	0,04
158	CD40 molecule, TNF receptor superfamily member 5 (CD40), transcript variant 1	−0,69	0,00
159	Signal peptide peptidase 3 (UNQ1887)	−0,69	0,00
160	MLCK protein (MLCK)	−0,70	0,04
161	Vacuolar protein sorting 24 homolog (*S. cerevisiae*) (VPS24), transcript variant 2	−0,70	0,02
162	LY6/PLAUR domain containing 1 (LYPD1), transcript variant 1	−0,71	0,02
163	Hypothetical protein FLJ31153 (FLJ31153), mRNA	−0,71	0,05
164	Fas apoptotic inhibitory molecule (FAIM), transcript variant 4	−0,71	0,05
165	ATR interacting protein (TREX1)	−0,72	0,03
166	EP300-interacting inhibitor of differentiation 3	−0,72	0,04
167	lin-7 homolog A (C, elegans) (LIN7A)	−0,73	0,02
168	Zeta-chain (TCR) associated protein kinase 70 kDa (ZAP70)	−0,73	0,05
169	Deleted in a mouse model of primary ciliary dyskinesia (RP11-529I10,4)	−0,74	0,04
170	N-ethylmaleimide-sensitive factor attachment protein, gamma (NAPG)	−0,74	0,02
171	Dynamin 2 (DNM2)	−0,74	0,00
172	Ribosomal protein L12 (RPL12)	−0,74	0,01
173	CD300 molecule-like family member g (CD300LG)	−0,75	0,00
174	4-Hydroxyphenylpyruvate dioxygenase	−0,75	0,04
175	Nuclease EXOG, mitochondrial	−0,76	0,01
176	Nuclear receptor coactivator 4 (NCOA4)	−0,76	0,02
177	Mitogen-activated protein kinase kinase kinase 14	−0,77	0,02
178	Chromosome 18 open reading frame 1 (C18orf1), transcript variant c2, mRNA	−0,77	0,04
179	Growth arrest-specific 2 (GAS2), transcript variant 2	−0,78	0,01
180	Transducin (beta)-like 1X-linked (TBL1X)	−0,79	0,03
181	Bone morphogenetic protein receptor, type IB (BMPR1B)	−0,79	0,03
182	Tropomodulin-2	−0,79	0,03
183	Calcium binding protein 39 (CAB39)	−0,81	0,03
184	Selectin P ligand (SELPLG)	−0,81	0,01
185	Neutrophil cytosolic factor 2 (65 kDa, chronic granulomatous disease, autosomal 2) (NCF2)	−0,82	0,02
186	Retinoic acid receptor, beta (RARB), transcript variant 2	−0,82	0,01
187	Potassium voltage-gated channel subfamily E member 1	−0,82	0,02
188	Interleukin-1 alpha	−0,82	0,01
189	Nucleoporin-like 1 (NUPL1), transcript variant 1	−0,82	0,01
190	HIG1 domain family, member 2A (HIGD2A)	−0,82	0,03
191	Pleiotropic regulator 1 (PRL1 homolog, *Arabidopsis*) (PLRG1)	−0,83	0,01
192	Coiled-coil domain containing 76, mRNA (cDNA clone MGC:87928 IMAGE:5104751), complete cds	−0,83	0,02
193	GTPase activating protein (SH3 domain) binding protein 1 (G3BP1), transcript variant 2	−0,84	0,03
194	Golgi SNAP receptor complex member 1 (GOSR1), transcript variant 1	−0,84	0,04
195	Phosphoglucomutase 2	−0,84	0,03
196	RAS-like, family 11, member B (RASL11B)	−0,86	0,05
197	Proteasome subunit alpha type 1	−0,86	0,04
198	MAP3K12-binding inhibitory protein 1	−0,89	0,00
199	Zinc finger, DHHC-type containing 11 (ZDHHC11)	−0,91	0,02
200	Moesin (MSN)	−0,92	0,01
201	Guanine nucleotide exchange factor DBS	−0,92	0,01
202	Chromosome 13 open reading frame 16 (C13orf16)	−0,92	0,04
203	Regulator of G-protein signaling 14 (RGS14)	−0,97	0,03
204	LSM4 homolog, U6 small nuclear RNA associated (*S. cerevisiae*) (LSM4)	−1,01	0,03
205	Fibronectin type III domain containing 4 (FNDC4)	−1,09	0,00
206	Myosin light chain kinase 2, skeletal muscle (MYLK2)	−1,12	0,03
207	HCG3 gene (HCG3)	−1,15	0,03
208	cAMP responsive element modulator (CREM), transcript variant 20, mRNA,	−1,31	0,03
209	TAF6 RNA polymerase II, TATA box binding protein (TBP) associated factor, 80 kDa (TAF6), transcript variant 1	−1,43	0,00
210	tec protein tyrosine kinase (TEC)	−1,76	0,03
211	Enolase 3 (beta, muscle) (ENO3)	−2,02	0,02
212	Chromosome 19 open reading frame 33 (C19orf33)	−2,40	0,02
213	26S proteasome non-ATPase regulatory subunit 7	−2,87	0,02

**Table 3:** The 14 autoantibodies building the classifier.

	Antibody directed against the following proteins	Ultimate ORF ID/catalog number
1	Retinoic acid receptor-beta (RARB) transcript variant 2	Hs~Ref:NM_016152.2~uORF:IOH36705~RFU:23189.6
2	Phosphoglucomutase 2-like 1 (PGM2L1)	Hs~MGC:BC059360.1~uORF:IOH29131~RFU:29573.42
3	Hepatitis B virus x interacting protein (HBXIP)	Hs~Ref:NM_006402.2~uORF:IOH40860~RFU:21469.91
4	Hypothetical protein MGC24103 (MGC24103)	Hs~MGC:NM_152576.1~uORF:IOH23047~RFU:19377.96
5	cAMP responsive element modulator (CREM), transcript variant 20, mRNA	Hs~Ref:NM_183012.1~uORF:IOH53457~RFU:0
6	Transient receptor potential cation channel subfamily M member 3	Hs~MGC:BC022454.2~uORF:IOH10977~RFU:4933.46
7	4-Hydroxyphenylpyruvate dioxygenase	Hs~Ref:NM_002150.1~uORF:IOH14718~RFU:30044.88
8	Chromosome 20 open reading frame 71 (C20orf71)	Hs~MGC:BC066354.1~uORF:IOH40076~RFU:14763.08
9	TNFSF10/APO2L/TRAIL/CD253 protein (native)	Hs~Ref:NP_003801.1~CAT_10409-HNAE-25~RFU:28.23
10	Kv channel interacting protein 4 (KCNIP4), transcript variant 1	Hs~Ref:NM_025221.4~uORF:IOH21934~RFU:27826.87
11	Exosome component 5 (EXOSC5)	Hs~MGC:BC007742.1~uORF:IOH6517~RFU:29914.87
12	Golgi SNAP receptor complex member 1 (GOSR1), transcript variant 1	Hs~Ref:NM_004871.2~uORF:IOH45920~RFU:29968.08
13	Chromosome 18 open reading frame 32 (C18orf32)	Hs~MGC:BC022357.1~uORF:IOH14149~RFU:28760.06
14	Proline-rich transmembrane protein 2 (PRRT2)	Hs~MGC:BC053594.1~uORF:IOH28968~RFU:10273.9
